# Tetrahedral framework nucleic acid nanomaterials reduce the inflammatory damage in sepsis by inhibiting pyroptosis

**DOI:** 10.1111/cpr.13424

**Published:** 2023-02-17

**Authors:** Xingyu Chen, Jiajun He, Yu Xie, Tianxu Zhang, Songhang Li, Yuxuan Zhao, Nan Hu, Xiaoxiao Cai

**Affiliations:** ^1^ State Key laboratory of Oral Diseases, National Clinical Research Center for Oral Diseases, West China Hospital of Stomatology Sichuan University Chengdu China; ^2^ Department of Stomatology First Medical Center, Chinese PLA General Hospital Beijng China

## Abstract

Sepsis is a highly lethal condition and is caused by the dysregulation of the body's immune response to infection. Indeed, sepsis remains the leading cause of death in severely ill patients, and currently, no effective treatment is available. Pyroptosis, which is mainly activated by cytoplasmic danger signals and eventually promote the release of the pro‐inflammatory factors, is a newly discovered programmed cell death procedure that clears infected cells while simultaneously triggering an inflammatory response. Increasing evidence indicates that pyroptosis participates in the development of sepsis. As a novel DNA nanomaterial, tetrahedral framework nucleic acids (tFNAs) characterized by its unique spatial structure, possess an excellent biosafety profile and can quickly enter the cell to impart anti‐inflammatory and anti‐oxidation effects. In this study, the roles of tFNAs in the in vitro model of macrophage cell pyroptosis and in the in vivo model of septic mice were examined, and it was found that tFNAs could mitigate organ inflammatory damage in septic mice, wherein they reduced inflammatory factor levels by inhibiting pyroptosis. These results provide possible new strategies for the future treatment of sepsis.

## Graphical abstract

tFNAs reduce the inflammatory damage in sepsis

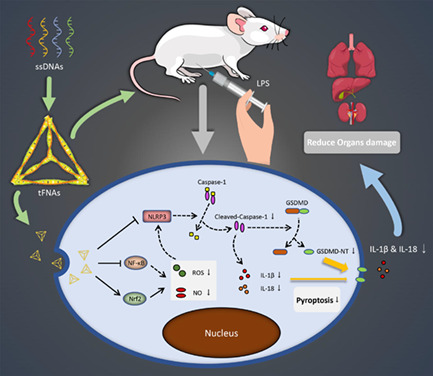

## 1 INTRODUCTION

Sepsis is a life‐threatening disease caused by dysregulated body's immune response to infection, which leads to wide‐ranging inflammatory reactions and serious complications.^1^, ^2^, ^3^ The survivors of sepsis often suffer from organ damage, and their quality of life is significantly decreased, as manifested by various physical dysfunctions and cognitive defects.^4^, ^5^ The occurrence of sepsis is mainly derived from bacterial infections, in which lipopolysaccharides (LPSs), as the main components of bacteria, are the main driver of mediating the severe inflammatory response in septic patients.^6^

It is believed that the pathological development of sepsis is closely related to the release of numerous inflammatory cytokines, which is triggered by pyroptosis.^7^, ^8^, ^9^, ^10^ After stimulation of the canonical pyroptosis pathway by pattern recognition receptors that recognize pathogen‐associated molecular patterns (PAMPs) or damage‐associated molecular patterns (DAMPs), inflammasomes (e.g., NLRP3, NACHT, LRR and PYD domain‐containing protein 3) within the cytoplasm are then activated and combined with apoptosis‐associated speck‐like protein containing CARD (ASC) to cleave pro‐caspase‐1 and form cleaved‐caspase‐1, which promotes the formation of activated inflammatory factors involving interleukin (IL)‐1β and IL‐18 by cleavage in pro‐IL‐1β and pro‐IL‐18. Meanwhile, cleaved‐caspase‐1 also dominates the formation of Gasdermin D N‐terminal (GSDMD‐NT), which composes pores on plasma membrane and mediates the extracellularly release of IL‐1β and IL‐18. Subsequently, by initiating an inflammatory response, the presence of excess amounts of inflammatory factors eventually leads to a wider range of cell pyroptosis.^11^, ^12^ In general, pyroptosis plays a positive role in the immunomodulatory process; however, when the host is undergoing severe infections like sepsis, it can be hyperactivated and exacerbates the wide‐ranging inflammation, which will eventually intensify the organ damage caused by sepsis.^8^ At present, the treatment strategy for sepsis is mainly anti‐infection treatment, and there is still no effective means of preventing sepsis or treating the resulting systemic organ inflammation. Therefore, reducing the inflammatory damage in sepsis through the inhibition of pyroptosis is now receiving growing attention as a potential new therapeutic approach.

Assembled from four specific single‐stranded fragments of DNA, the tetrahedral framework nucleic acids (tFNAs) are novel nucleic acid nanomaterials with a tetrahedral spatial structure, which imparts them with excellent biological properties, including enhanced cell endocytosis properties and a superior tissue permeability, thereby rendering them suitable for application in disease treatment, drug delivery, biosensing and other fields.^13^, ^14^, ^15^, ^16^, ^17^, ^18^, ^19^ Moreover, previous studies have shown that tFNAs exhibit excellent anti‐inflammatory antioxidant capacities and good biosafety profiles in the treatment of inflammatory diseases, which is mainly achieved by their regulation of the nuclear factor kappa‐light‐chain‐enhancer (Nf‐κB) pathway.^20^ In addition, tFNAs can regulate the polarization of macrophages by inhibiting pro‐inflammatory M1 types and promoting the scavenging of intracellular reactive oxygen species (ROS).^16^, ^21^, ^22^ It should be noted that abnormal intracellular ROS levels and activation of the NF‐κB pathway can raise the expression of pro‐inflammatory factors and the NLRP3 inflammasome, thereby promoting pyroptosis and triggering a strong inflammatory response.^23^, ^24^, ^25^ Therefore, due to the excellent anti‐inflammatory and antioxidant abilities of tFNAs, they are expected to inhibit sepsis‐related inflammatory responses, revealing a novel strategy for organ protection during sepsis.

In this study, we establish an in vitro model of RAW264.7 macrophage pyroptosis and an in vivo model of septic BALB/c mice to explore the positive role of tFNAs in regulating cell pyroptosis to reduce the inflammatory response and mitigate multi‐organ damage in sepsis.

## 2 MATERIALS AND METHODS

### 2.1 Generation of the tFNAs

Based on the Watson–Crick base‐pairing principles, tFNAs nanomaterial was self‐assembled from four single‐stranded sequence‐specific DNA (ssDNA) fragments in equimolar quantities (1 μM), as outlined in Table 1 (Sangon Co., Ltd.), using TM buffer (composed of 50 mM MgCl_2_ and 10 mM TrisHCl (pH 8.0) solutions) and heating at 95°C for 10 min followed by rapid cooling to 4°C for 20 min. The obtained tFNAs were further stored in a fridge at 4°C until required for use.

**TABLE 1:** The sequence of ssDNAs.

ssDNAs	Base sequence (5′‐3′)
S1	ATTTATCACCCGCCATAGTAGACGTATCACCAGGCAGTTGAGACGAAC ATTCCTAAGTCTGAA
S2	ACATGCGAGGGTCCAATACCGACGATTACAGCTTGCTACACGATTCAGACTTAGGAATGTTCG
S3	CTACTATGGCGGGTGATAAAACGTGTAGCAAGCTGTAATCGACGGGAAGAGCATGCCCATCC
S4	ACGGTATTGGACCCTCGCATGACTCAACTGCCTGGTGATACGAGGATGGGCATGCTCTTCCCG
Cy5‐S1	Cy5ACGGTATTGGACCCTCGCATGACTCAACTGCCTGGTGATACGAGGATGGGCATGCTCTTCCCG

### 2.2 Authentication and characterization of the tFNAs

After the successful synthesis of tFNAs, their molecular weights were determined using 8% polyacrylamide gel electrophoresis (PAGE), and the purity of the tFNAs was evaluated by the capillary electrophoresis. For intuitive observations regarding the spatial nanostructures of the tFNAs, transmission electron microscopy (TEM, Hitachi Ltd.) and atomic force microscopy (AFM, Shimadzu) were used. The particles sizes and zeta potential of the tFNAs were measured by dynamic light scattering (DLS, Nano ZS).

### 2.3 Cellular experiments

The murine macrophage (Raw264.7) was cultured in high‐glucose Dulbecco's Modified Eagle Medium (DMEM) complete medium containing 10% foetal bovine serum (HyClone) and 1% penicillin–streptomycin solution (HyClone) in an incubator at 37°C containing 5% CO_2_ under a humidified atmosphere. Subsequently, the cells were divided into three groups, namely the blank, the control (ctrl), and the tFNAs group. While no intervention was performed for the blank group, the cells in the tFNAs group were pre‐treated with tFNAs (250 nM) for 2 h, and then the cells in both the ctrl and tFNAs groups were administrated with 1 μg/mL LPS solution from *Escherichia coli* O127:B8, (Sigma‐Aldrich) and 5 mmol/L adenosine triphosphate (ATP, Sigma‐Aldrich) for 4 h to induce pyroptosis.

### 2.4 Animal experiments

Eighteen BALB/c mice aged 8 weeks (male, 20–22 g body mass) were randomly separated into three experimental groups (blank, ctrl and tFNAs groups) and allowed free access to water and food under standard environment conditions (24–26°C, 50% humidity, 12/12 h light–dark cycle). All animal experiments were conducted according to the ethical guidelines outlined by the Animal Ethics Committee of West China College of Stomatology, Sichuan University, and the mice were allowed to acclimatize for 1 week prior to intervention. To explore the multi‐organ inflammatory protection effects of tFNAs in septic mice, the saline (0.9%, 200 μL) or tFNAs (1 μM, 200 μL) solutions was intravenously injected into the tail vein of the mice on day 1 and day 3. Subsequently, the mice of the ctrl and tFNAs groups were intraperitoneally injected with LPS (10 mg/kg) on day 4 to induce sepsis. After 12 h, the whole blood samples were collected for cytometric measurements, and were subjected to centrifugation to obtain the corresponding serum samples for evaluation the degree of IL‐1β and IL‐18 production by the cytokines. In addition, the livers, kidneys and lungs of euthanized mice were harvested to perform haematoxylin and eosin (H&E) stainings for evaluation of the inflammatory damage and immunofluorescence staining to determine the expression of IL‐1β and IL‐18.

### 2.5 Uptake of tFNAs by macrophage cells

To examine the process by which the tFNAs enter into macrophages, Cy5‐labelled S1 (Cy5‐S1) with a red fluorescence signal was used instead of S1 to synthesize Cy5‐labelled tFNAs for follow‐up tests. Initially, RAW264.7 cells were treated with the Cy5‐labelled tFNAs for 1, 2, 3 and 4 h and were then collected for detection. The ratios of the number of cells exhibiting Cy5 fluorescence to the total number of cells were determined using a flow cytometer (CytoFLEX, Beckman Coulter Inc.). In addition, for visual inspection of the content of intracellular tFNAs, images of the Cy5‐labelled tFNAs dispersed in the cells were captured using confocal microscopy (Olympus).

### 2.6 Cell viability assays

The cell viability was determined using the commercially available Cell Counting Kit 8 (MedChemExpress) to evaluate the cytotoxic nature of the tFNAs. RAW264.7 cells were cultured at a density of 5000 cells per well in a 96‐wells plate and were pre‐incubated at 37°C in a 5% CO_2_ incubator for 12 h. After pre‐treatment with different concentrations of tFNAs (i.e., 125, 250, 375, or 500 nM) for 12 h, the cells were incubated the CCK‐8 solution (10 μL) for 1 h. The optical density (OD) values at 450 nm were recorded to indicate the cell viability.

### 2.7 LDH level detection assays

The lactate dehydrogenase (LDH) activity in the supernatant was used to evaluate the plasma membrane integrity and the degree of cell death under pyroptosis. Cells were cultured in 96‐wells plate at a density of 5000 cells per well, and after the intervention mentioned in Section 2.3, the supernatants of the different groups were collected and subjected to centrifugation (300× g for 5 min) to remove all cellular debris. The LDH measurements were then conducted using a CytoTox 96 Non‐Radioactive Cytotoxicity Assay kit (Promega), according to the manufacturer's protocol.

### 2.8 Western blot analysis

The RAW264.7 cells were seeded in a 6‐well plate (10^6^ cells per well). After the treatment with the tFNAs and the addition of LPS and ATP, protein samples were obtained from the different groups using whole‐protein extraction kits (KeyGen Biotech Co., Ltd.). The primary antibodies (i.e., anti‐β‐actin, anti‐NF‐κB‐p65, anti‐NF‐κB‐p‐p65, anti‐Nrf2, anti‐NLRP3, anti‐GSDMD, anti‐GSDMD N‐terminal, anti‐caspase‐1, anti‐cleaved caspase‐1, anti‐IL‐1β and anti‐IL‐18) were purchased from Abcam. After incubation for 24 h with the primary antibodies (dilution ratio according to the manufacturer's protocol), the protein samples were incubated with secondary antibodies (1:2000) over a period of 1 h, wherein they were washed 3 times (5 min per wash) with Tris Buffered Saline with Tween (TBST) solution (1×) between each step. Enhanced chemiluminescence (ECL) detection (Bio‐Rad) was performed to detect the protein bands, and the semi‐quantitative analysis of the results was carried out using the ImageJ software.

### 2.9 Measurement of cytokine production using the enzyme‐linked immunosorbent assay (ELISA) approach

To verify the anti‐inflammatory abilities of the prepared tFNAs, RAW267.4 cells were cultured in 6‐well plates (10^6^ cells per well) and treated as described in Section 2.3. The cell supernatants were then collected and divided into different groups. The serum samples were obtained from the mice specimens 12 h after the addition of LPS. The secretion of inflammatory cytokines (IL‐1β and IL‐18) from the cells and their concentrations in the serum were measured using ELISA kits (MultiSciences) according to the manufacturer's instructions.

### 2.10 Detection of the ROS levels

The levels of intracellular ROS present in the RAW264.7 cells were determined using a DCFH‐DA assay kit (Beyotime). The cells were cultured in 6‐well plates at a density of 10^6^ cells per well and pre‐treated with or without 250 nM tFNAs. After 4 h stimulation by LPS and ATP, the cells in the different groups were rinsed with phosphate‐buffered saline (PBS), followed by incubation with a DCFH‐DA probe for 20 min. Subsequently, fluorescence images of the intracellular ROS (green) were obtained using an optical microscope (Leica), and the specific fluorescence intensities were analysed using the ImageJ software.

### 2.11 Detection of the nitric oxide (NO) levels

To measure the NO levels, present in the RAW264.7 cells, a Griess Assay Kit (Beyotime) was employed. For this purpose, the RAW264.7 cells were cultured in a 12‐well plate (10^5^ cells per well), wherein they were grouped and treated as described in Section 2.3, then the supernatants of the different groups were collected for subjected to centrifugation (300× g for 5 min) to remove all cellular debris. The obtained supernatants were subsequently transferred to a 96‐well plate (50 μL per well), and Griess assay kit solutions 1 and 2 (50 μL each) were added as per the manufacturer's instructions. The levels of NO were calculated by measuring the absorbance at 540 nm.

### 2.12 Statistical analysis

The GraphPad Prism software version 8.0.2 was used for statistical analysis. The statistical method employed for comparison between the groups involved Student's *t*‐test and one‐way analysis of variance (ANOVA). All quantitative data were presented as the mean ± standard deviation (*n* ≥ 3).

## 3 RESULTS AND DISCUSSION

### 3.1 Preparation of the tFNAs

**FIGURE 1:**
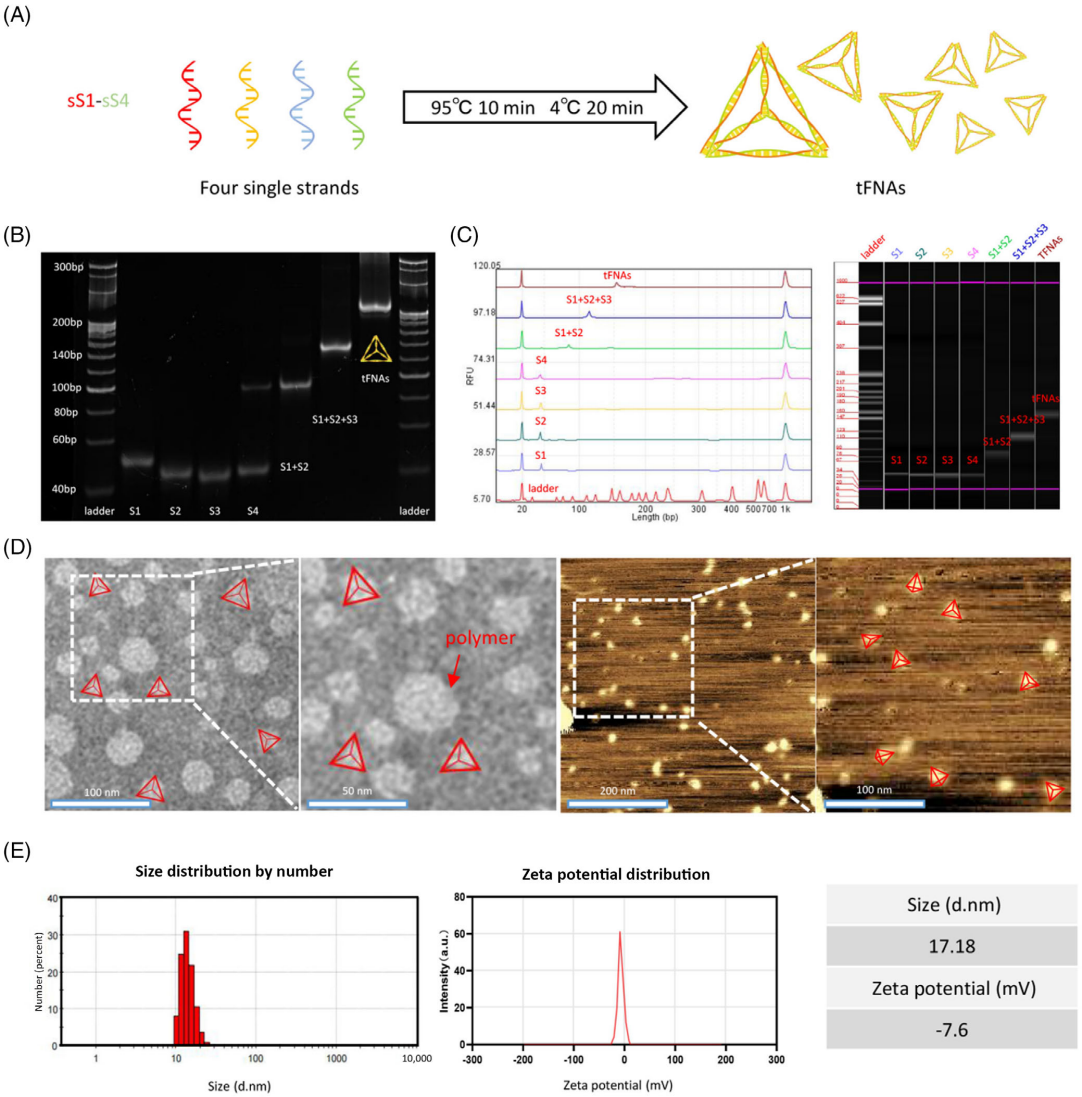
Synthesis and characterization of the tetrahedral framework nucleic acids (tFNAs). (A) Schematic representation of the synthesis process employed to obtain the tFNAs. (B) Confirmation of the successful synthesis of the tFNAs by polyacrylamide gel electrophoresis (Lane 1: ladder; Lane 2: S1; Lane 3: S2; Lane 4: S3; Lane 5: S4; Lane 6: S1 + S2; Lane 7: S1 + S2 + S3; Lane 8: tFNAs; Lane 9: ladder). (C) Capillary electrophoresis results demonstrating successful preparation of the tFNAs. (D) TEM image of the tFNAs. (Scale bar: 100 nm) and the corresponding atomic force microscopic image of the tFNAs (scale bar: 200 nm). (E) Size distribution and zeta potential results for the tFNAs measured by dynamic light scattering.

Four ssDNA segments (S1−S4) composed of the specific nucleic acid sequences outlined in Table 1 were added to the TM buffer solution and conducted as described in Section 2.1, which resulted in their self‐assembly to generate a stable tetrahedral structure (Figure 1A). The successful synthesis of the tFNAs was confirmed using PAGE and capillary electrophoresis (Figure 1B,C). Subsequent imaging using TEM and AFM revealed the tetrahedron‐like structures of the tFNAs, as indicated by the triangular nanoparticles that are labelled in Figure 1D. DLS measurements indicated that the average size of the tFNAs particle was ∼17 nm, while the average zeta potential value was determined to be −7.6 mV (Figure 1E).

### 3.2 Cellular uptake of the tFNAs by macrophages

**FIGURE 2:**
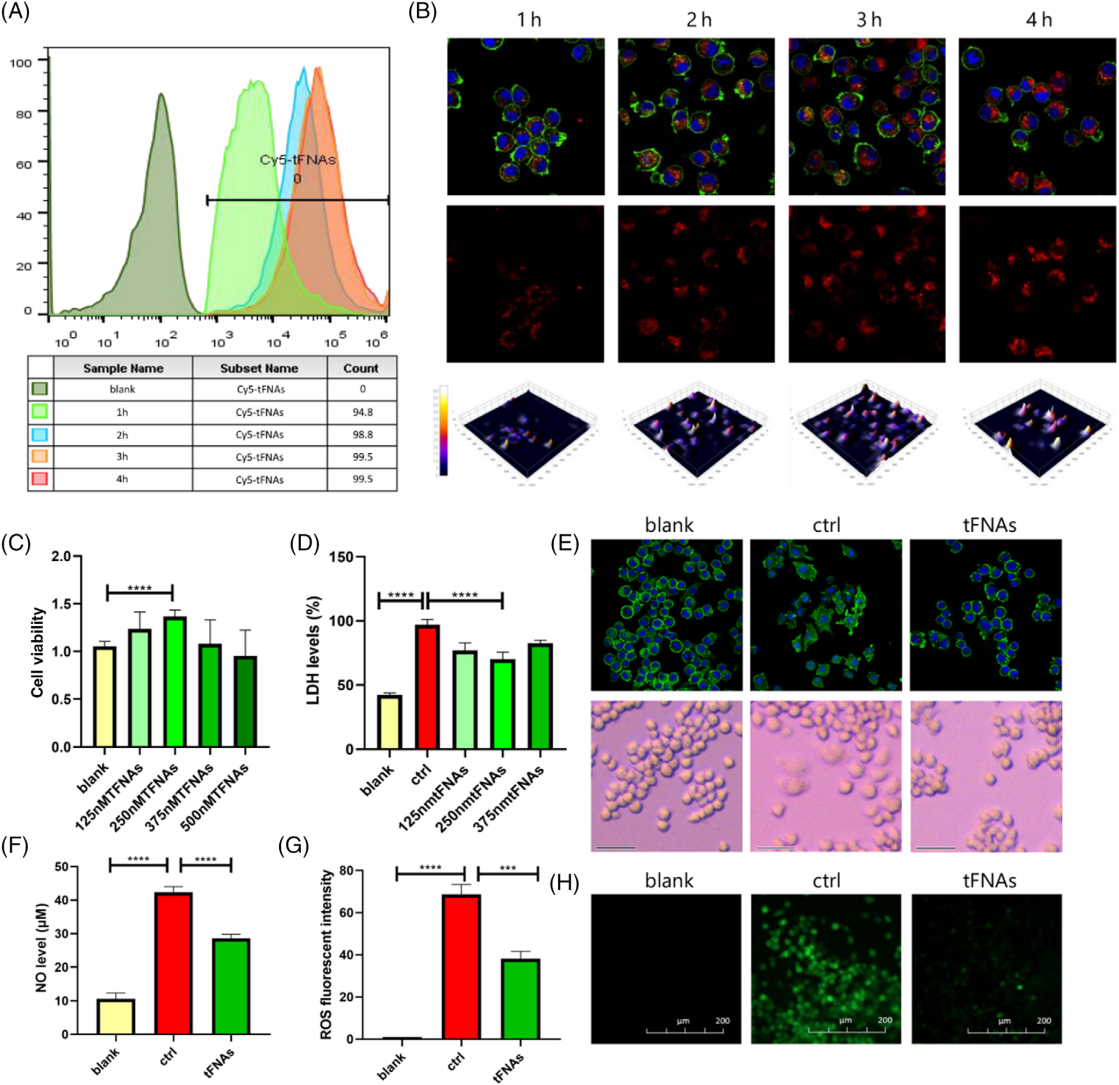
Inhibition of changes in the integrity of the RAW267.4 in the pyroptosis state by action of the tetrahedral framework nucleic acids (tFNAs). Reduction of the reactive oxygen species (ROS) and nitric oxide (NO) levels is also shown. (A) Quantitative detection and analysis of the intracellular tFNAs levels using flow cytometry. (B) Cellular uptake of the Cy5‐tFNAs, as determined by immunofluorescence staining (Blue: nucleus; Red: Cy5‐labelled tFNAs; Green: cytoskeleton). (C) The cell viability of the RAW267.4 cells was evaluated after exposure to tFNAs of different concentrations. (D) Lactate dehydrogenase levels in the supernatant under pyroptosis conditions after different treatment regimes. (E) Cellular morphology under confocal microscopy and light microscopy. (F) NO levels of the RAW264.7 cells after different treatment regimes. (G) Fluorescent intensities representing intracellular ROS levels after different treatment regimes. (H) Imaging of the intracellular ROS levels (Green: ROS; Scale bar: 200 nm). Data are presented as mean ± standard deviation (*SD*) (*n* ≥ 3). Statistical analysis: *p < 0.05, **p < 0.01, ***p < 0.001

Previous studies confirmed that the tFNAs exhibit excellent cell‐entry properties due to their unique spatial structures.^16^ To probe the time required for the tFNAs to enter the RAW267.4 cells and reach their maximum entry efficiency, S1 was modified by Cy‐5, and Cy‐5‐labelled tFNAs were prepared and added to the RAW267.4 cells (250 nM Cy‐5‐labelled tFNAs). In general, the cells were treated with Cy5‐labelled tFNAs for 1, 2, 3 and 4 h, and then collected to quantify the degree of Cy‐5 fluorescence in the cells using flow cytometry. As shown in Figure 2A, the highest fluorescence intensities associated with tFNAs presence in the cells were reached after ∼2–3 h of incubation. Additionally, confocal microscopy was used to examine the intracellular content of the Cy‐5‐labelled tFNAs after 1, 2, 3 and 4 h of incubation (Figure 2B). It was found that after 1 h, the cells showed only weak fluorescence; however, after 2–3 h, the fluorescence intensity increased significantly. Hence, the tFNAs pre‐treatment time was set to 2 h. Cell viability assays showed that under normal conditions, a tFNAs concentration of 250 nM effectively promoted cell proliferation (Figure 2C), which is in agreement with previous studies,^21^ thereby confirming the excellent biocompatibility of the tFNAs at this concentration.

### 3.3 Maintenance of the plasma integrity under pyroptosis conditions by action of tFNAs

Unlike pyroptotic cells, cells in the apoptotic state maintain their plasma membrane integrity and induce a non‐inflammatory consequence by not releasing inflammatory mediators.^26^ However, when pyroptosis occurs, GSDMD‐NT, which is generated from the cleavage of Gasdermin D by cleaved‐caspase‐1, accumulates in the cell membrane and forms pores, resulting in water inflow and potassium ion outflow. As a result, the plasma membrane potential becomes unstable, the cells gradually expand and the cell membrane integrity is destroyed by cell rupture.^27^, ^28^, ^29^ Such a process is often rapid and uncontrollable in pathological states, resulting in the excessive activation of pyroptosis and exacerbation of the inflammatory response associated with LPS‐induced sepsis.^10^, ^30^, ^31^

To determine whether pre‐treatment with the tFNAs affected the membrane integrity under pyroptosis conditions, the macrophages were separated into three intervention groups, as described in Section 2.3. The blank and the ctrl groups were not subjected to any pre‐treatment, while tFNAs (250 nM) were added to the tFNAs group 2 h in advance. As can be seen in the confocal microscopy images presented in Figure 2E, the cell membrane (green) integrity of the ctrl group was disrupted and the cells presented an enlarged state; however, the condition of the cells in the tFNAs group were similar to that of the cells in the blank group, wherein all cells were uniform in size and the plasma membrane was intact. Comparable results were observed using optical microscopy to image the membranes of the different sample groups. Furthermore, based on the Cytotox96 test results, it was found that the LDH level in the supernatant of the tFNAs group was significantly lower than that of the ctrl group (Figure 2D), indicating that tFNAs pre‐treatment effectively maintained the integrity of the cell membrane under pyroptosis. More specifically, tFNAs pre‐treatment mitigated the impacts of pyroptosis on macrophages, reduced the degree of cytotoxicity and showed the potential on alleviating the wide‐ranging inflammatory response caused by the release of massive inflammatory factors during hyperactivated pyroptosis.

### 3.4 Decrease of the LPS‐induced ROS and intracellular NO contents by action of the tFNAs

When immune cells are stimulated by LPS, excessive amounts of NO and endogenous ROS are produced in the cells, which can cause cell damage and further promote activation of the NLRP3 inflammasome, thereby mediating cell pyroptosis.^23^, ^24^, ^32^ In addition, a normal amount of NO is crucial to ensuring the health of our bodies; however, the abnormal generation of NO can cause the dysfunction of multiple systems and can even lead to tissue damage.^33^ Therefore, reducing intracellular NO levels whilst intervening against excessive oxidative stress can be achieved using antioxidants, and this has been considered a possible therapeutic strategy for sepsis.^6^, ^34^, ^35^, ^36^

Thus, the roles of the tFNAs in scavenging excessive intracellular ROS and reducing the intracellular levels of NO were evaluated. More specifically, cells were treated as described in Section 2.3 and subsequently, the ROS clearance effect imparted by the tFNAs was determined by fluorescence microscopy (Figure 2H). Under the stimulation of LPS, large amounts of ROS were produced in the macrophages (Figure 2G), as evidenced by a strong fluorescence intensity. Pre‐treatment with the tFNAs significantly reduced the intracellular ROS levels, thereby suggesting that they exhibited a certain antioxidant effect. Moreover, the intracellular NO levels of the cells in the blank, ctrl and tFNAs groups were determined using a Griess detection kit. It was found that both the tFNAs and ctrl groups contained significantly higher levels of intracellular NO than the blank groups after LPS + ATP stimulation; however, the level of intracellular NO in the tFNAs group was significantly decreased compared with that of the ctrl group (Figure 2F), which indicates that pre‐treatment with the tFNAs can block NO overproduction to prevent a possible severe inflammatory response. Although the above result reveals the potential of tFNAs as antioxidants, the mechanism by which the tFNAs effect the generation of the overproduced NO and the excessive ROS, remains unclear. Further exploration is therefore needed in this field in the future.

### 3.5 Suppression of pyroptosis‐ and inflammation‐related protein expression by the tFNAs

Upon stimulation by LPS, the toll‐like receptors on the surfaces of macrophages are activated and transmit signals to the nucleus via several transcription factors including the NF‐κB family, which is known to promote the expression of various genes, such as pro‐IL‐1β and pro‐IL‐18.^34^, ^37^ Under further stimulation by ATP and the high levels of intracellular ROS, the NLRP3 inflammasome is activated and over‐expressed to combine with ASC and cleave pro‐caspase‐1, triggering the activation of the canonical pyroptosis pathway.^8^, ^23^, ^38^ As a crucial pathway against oxidative stress, the high expression of nuclear factor‐erythroid 2 related factor 2 (Nrf2) is closely associated with the anti‐oxidative capacity of cells. Thus, to explore whether the anti‐oxidative role of tFNAs is related to the Nrf2 pathway, the protein expression of Nrf2 was detected in the three experimental groups.^39^, ^40^

**FIGURE 3:**
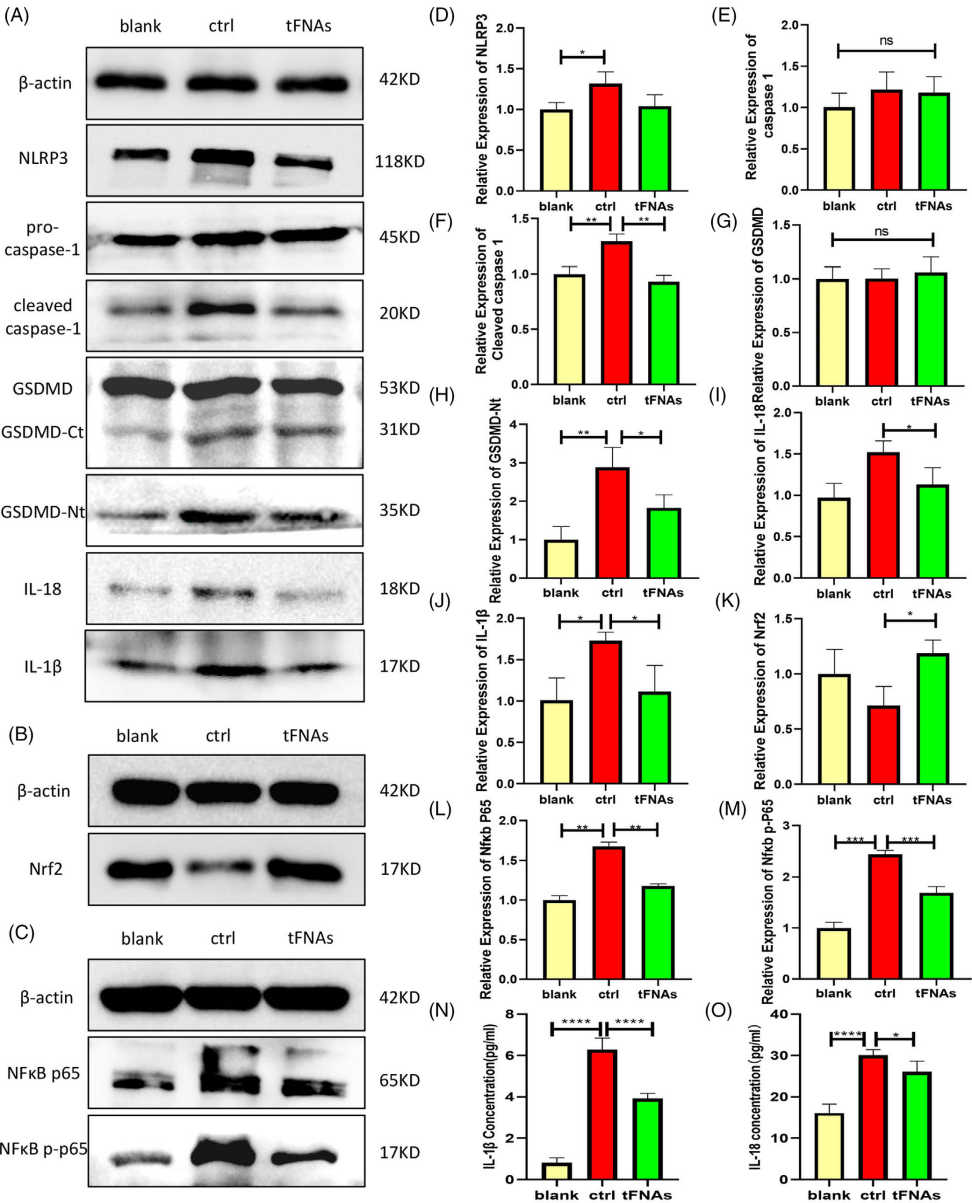
Modulation of the pyroptosis and inflammatory signalling molecules. (A–M) Western blotting results and relative expression levels of NLRP3, pro‐caspase‐1, cleaved‐caspase‐1, GSDMD, GSDMD‐Ct, GSDMD‐Nt, IL‐18, IL‐1β, NF‐κB p65, NF‐κB p‐p65 and Nrf2. (I–N) Secretion of inflammatory cytokines including IL‐1β and IL‐18 detected by enzyme‐linked immunosorbent assay. Data are presented as mean ± standard deviation (*SD*) (*n* ≥ 3). Statistical analysis: *p < 0.05, **p < 0.01, ***p < 0.001

To verify regulation of the inflammatory oxidative stress and the pyroptotic pathway by the tFNAs, expression of the related proteins was determined by Western blotting (Figure 3A–C). As expected, the expression of NLRP3 in the tFNAs group was significantly lower than that in the ctrl group (Figure 3D), indicating that tFNAs pre‐treatment reduced the LPS + ATP‐induced activation of cellular inflammasomes. Although there was no significant difference in caspase‐1 expression, the cleaved‐caspase‐1 expression levels indicated that the cleavage activation of caspase‐1 is reduced because of the low expression levels of NLRP3 in the tFNAs group (Figure 3E,F). This also accounts for the significantly lower levels of GSDMD‐NT (Figure 3H) and the reduced expression of IL‐18 and IL‐1β (Figure 3I,J) in the tFNAs group. In addition, the protein expression results obtained for NF‐κB p65 (Figure 3L) and NF‐κB p‐p65 (Figure 3M) indicated that tFNAs pre‐treatment indeed reduced the inflammatory activation of macrophages caused by pathogenic stimuli, whilst also improving the anti‐inflammatory ability of the immune cells. The high expression of the Nrf2 protein (Figure 3K) in the tFNAs group also suggested that such pre‐treatment enhanced the ability of the immune cells to resist oxidative stress. Moreover, the results of the ELISA tests carried out on the cell supernatants also yielded a lower content of extracellular IL‐18 and IL‐1β in the tFNAs group compares to the ctrl group (Figure 3N,O). The above experimental results therefore confirmed our hypothesis.

### 3.6 Reduction LPS‐induced systemic inflammation damage in septic mice by action of the tFNAs

**FIGURE 4:**
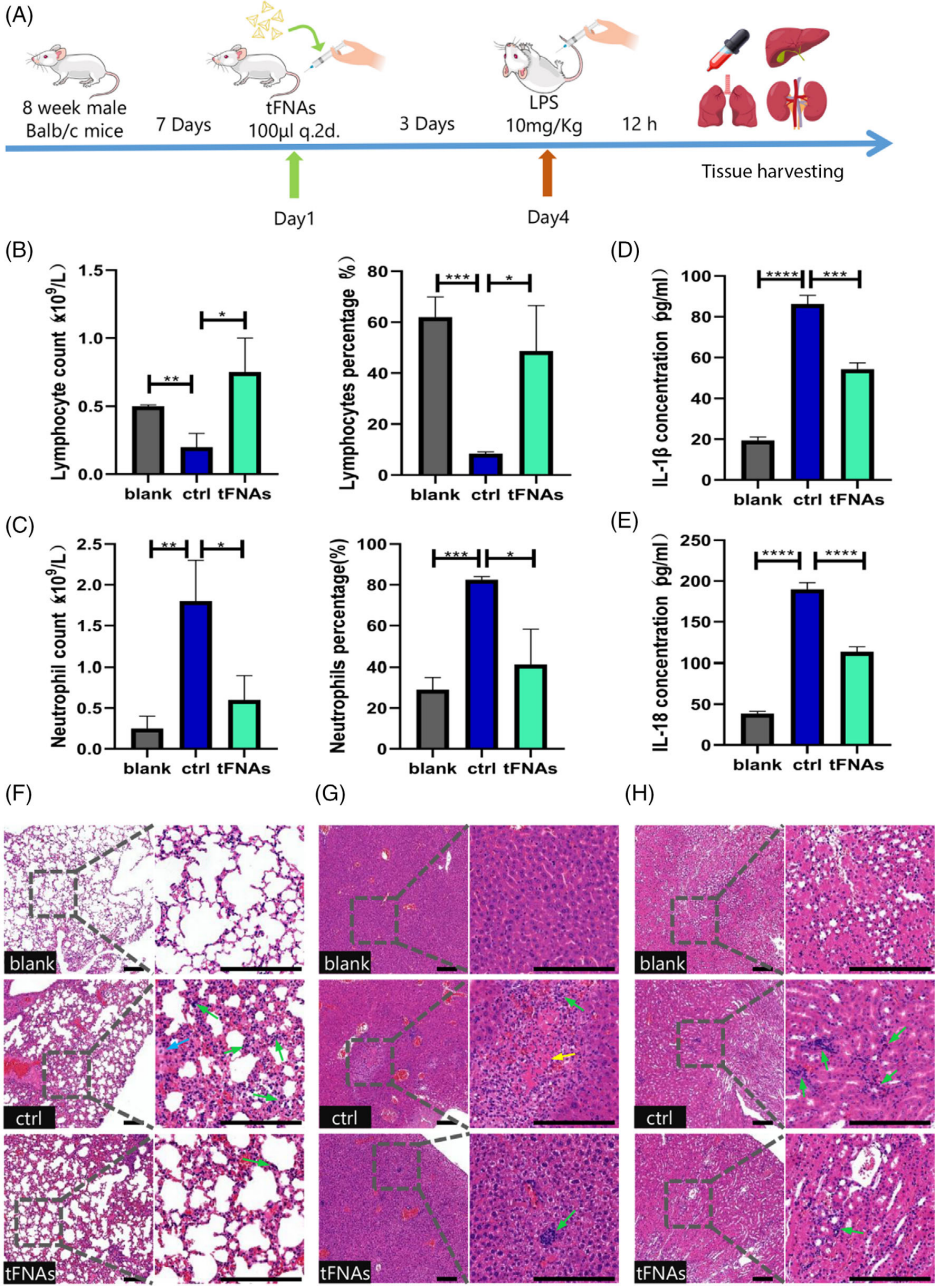
Reduction of lipopolysaccharide‐induced systemic inflammation damage in septic mice by the tetrahedral framework nucleic acids (tFNAs). (A) Schematic diagram outlining pre‐treatment with the tFNAs and the establishment of an in vivo animal system. (B,C) Cytometric results for the lymphocytes and neutrophils in the different treatment groups. (D,E) Enzyme‐linked immunosorbent assay detection of inflammatory cytokines, including IL‐1β and IL‐18 in the serum. (F–H) H&E staining images of the lung, liver and kidney specimens (scale bar: 200 μm; Green arrows: inflammatory cell infiltration; yellow arrows: haemorrhage; blue arrows: necrotic cells). Data are presented as mean ± standard deviation (*SD*) (*n* ≥ 3). Statistical analysis: *p < 0.05, **p < 0.01, ***p < 0.001

Patients with LPS‐induced sepsis often suffer from wide‐ranging inflammatory damage and multiple organ dysfunction due to the massive degree of inflammation.^2^, ^41^ In this context, neutrophils play a crucial role in inducing and activating inflammatory responses while lymphocytes protect multiple organs from inflammatory damage. Therefore, the ratio of neutrophils to lymphocytes is a marker of the inflammatory response and immune balance, wherein a high ratio correlates with severe inflammation.^42^ To verify the in vivo protective effect of tFNAs against inflammation, we constructed a mouse sepsis model by the intraperitoneal injection of LPS (10 mg/kg). More specifically, on days 1 and 3, 1 μM tFNAs solution (200 μL) or an equivalent amount of the saline (for the blank and ctrl groups) was injected into the tail vein of each mouse. On day 4, LPS (10 mg/kg) was injected intraperitoneally (for the ctrl and tFNAs groups), while an equivalent amount of saline was intraperitoneally injected into the mice in the blank group. Blood, serum, liver, kidney, and lung samples were collected from all mice after 12 h, as outlined schematically in Figure 4A.

Following cytometric testing, the total number and percentage of lymphocytes and neutrophils in the ctrl group were found to be significantly lower and higher than those in the tFNAs group, respectively (Figure 4B,C), indicating that the mice in the tFNAs group exhibited a less severe inflammatory reaction. Meanwhile, the ELISA test showed that the levels of IL‐1β and IL‐18 in the serum of the LPS‐induced septic mice after tFNAs pre‐treatment were significantly reduced compared to those in the ctrl group (Figure 4D,E). The above results therefore show that pre‐treatment with tFNAs can effectively reduce the expression levels of both cytokines during sepsis.

**FIGURE 5:**
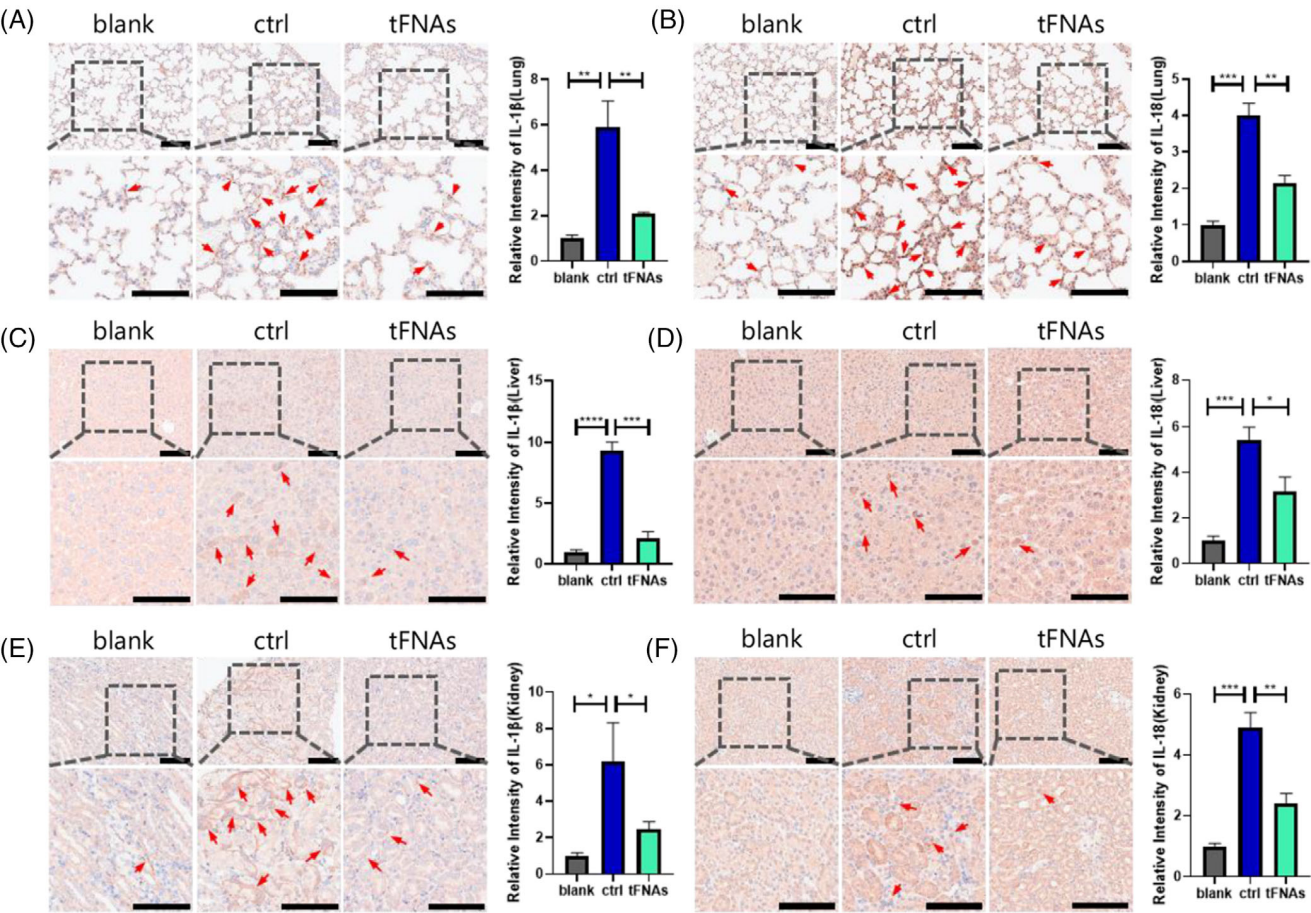
Reduced production of inflammatory cytokines in multiple organs under the action of the tetrahedral framework nucleic acids (tFNAs). (A) IL‐1β expression in the lung, as revealed by immunohistochemical staining (IHC) and statistical analysis. (B) IL‐18 expression in the lung and statistical analysis. (C) IL‐1β expression in the liver and statistical analysis. (D) IL‐18 expression in the liver and statistical analysis. (E) IL‐1β expression in the kidney and statistical analysis. (F) IL‐18 expression in the kidney and statistical analysis (Scale bar: 100 μm; Arrows: inflammatory cytokines). Data are presented as mean ± standard deviation (*SD*) (*n* ≥ 3). Statistical analysis: *p < 0.05, **p < 0.01, ***p < 0.001

To explore the anti‐inflammatory ability of tFNAs on various organs, the infiltration of neutrophils was observed in the liver, kidney and lung using H&E staining (Figure 4F–H). The resulting images revealed that the tissues of the ctrl group exhibited higher degrees of neutrophil infiltration than with the tFNAs group, and the manifestations associated with tissue damage, such as cell necrosis and acute haemorrhage, were correspondingly increased. Finally, the distributions of the inflammatory factors IL‐18 and IL‐1β were evaluated in these organs by means of immunohistochemistry staining (Figure 5A–F). It was found that in all organs, the expression of these inflammatory factors were significantly suppressed in the tFNAs group compared with the ctrl group, which further confirms the protective effect of tFNAs against inflammation in LPS‐induced septic mice. These findings indicate that tFNAs pre‐treatment may improve the body's tolerance to inflammatory responses and reduce the degree of tissue damage during the state of inflammatory stress.

## 4 CONCLUSION

In summary, we successfully synthesized a nucleic acid nanomaterial with a tetrahedral spatial structure (tFNAs). The results of the above experiments confirmed the protective effects of tFNAs against macrophages under LPS pathogen stimulation, and their preventive effect on reducing the inflammatory response in septic mice was also demonstrated. This process was considered to take place due to inhibition of the Nf‐κB pathway, a reduction in the gene expression associated with pro‐inflammatory factors, promotion of the expression of Nrf2, and the scavenging of excess intracellular ROS and NO. Subsequently, the activation and expression of NLRP3 were decreased and followed by the inhibition of pyroptosis and reduction of the degrees of activation and release of inflammatory factors, ultimately mitigating the inflammatory response. Due to the discovery of a close relationship between sepsis and pyroptosis, the regulation of pyroptosis to reduce the inflammatory response appears to be promising as a novel strategy to treat sepsis. However, the specific mechanism of pyroptosis is not yet fully understood, and the role of tFNAs in regulating pyroptosis has still to be determined relative to other types of inflammasomes, such as caspase and the gasdermin family proteins. Furthermore, it is necessary to determine whether the inflammatory protective effect of tFNAs in septic mice can be reproduced in humans. Nevertheless, as a nucleic acid nanomaterial with good biosafety profile, the anti‐oxidative and anti‐inflammatory effects of tFNAs and their ability to regulate cell pyroptosis indicate the potential of this system to treat inflammation‐ and pyroptosis‐related diseases. Moreover, due to their ease of editing and their rapid penetration properties, tFNAs have the potential for use as carriers to load small drug molecules, micro‐RNA and aptamers, for further applications.^43^ In the future, we will focus on the multifaceted applications of these novel nucleic acid nanomaterials and will explore in detail the underlying mechanisms that lead to their various biological capabilities.

## AUTHOR CONTRIBUTIONS

Xingyu Chen conceived and designed the research, performed the experiments and data collection. Jiajun He and Yu Xie helped with the data analysis. Nan Hu and Xiaoxiao Cai supervised the project, and reviewed, edited and finalized the manuscript.

## CONFLICT OF INTEREST STATEMENT

The authors declare no conflicts of interest.

## Data Availability

The data that support the findings of this study are available from the corresponding author upon reasonable request.
